# In vitro accuracy of digital and conventional impressions in the partially edentulous maxilla

**DOI:** 10.1007/s00784-022-04598-4

**Published:** 2022-07-01

**Authors:** Moritz Waldecker, Stefan Rues, Junior Sinclair Awounvo Awounvo, Peter Rammelsberg, Wolfgang Bömicke

**Affiliations:** 1 Department of Prosthetic Dentistry, Heidelberg University Hospital, University of Heidelberg, Im Neuenheimer Feld 400, 69120 Heidelberg, Germany; 2 Institute of Medical Biometry, Heidelberg University Hospital, University of Heidelberg, Heidelberg, Germany

**Keywords:** Digital impression, Intraoral scan, Conventional impression, Complete arch, Partially edentulous

## Abstract

**Objectives:**

This in vitro study compared the dimensional accuracy of conventional impressions (CI) with that of digital impressions (DI) in a partially edentulous maxilla. DIs were made by two intraoral scanners, Omnicam (OC) and Primescan (PS).

**Materials and methods:**

CI and both intraoral scanners were used to take 30 impressions of two identical reference models. CIs were poured with type 4 gypsum and the saw-cut models were digitized. The reference models simulated a maxilla with six prepared teeth that accommodated a cross-arch fixed partial denture. Center points of five precision balls and center points at the margin level of each prepared tooth were used to detect changes in dimensions and tooth axis between the reference model and the scans.

**Results:**

For DI, the largest deviations (176 µm for OC and 122 µm for PS) occurred over the cross-arch. For CI, the largest deviation (118 µm) occurred over the anterior segment. For shorter distances up to a quadrant, DI was superior to CI. For longer scan distances, DI was comparable (2 sextant and anterior segment) or inferior (cross-arch) to CI. Vertical and tooth axis deviations were significantly smaller for CI than for DI (*p* < 0.001).

**Conclusions:**

The impression method affected the impression accuracy of a partially edentulous maxilla with prepared teeth. DI is recommended for scans up to a quadrant. Larger scan volumes are not yet suitable for fabricating a fixed partial denture because of the high scatter of accuracy values.

**Clinical relevance:**

In contrast to conventional impressions, digital impressions lead to comparable or better results concerning scans up to a quadrant. Consequently, for larger scan volumes, several smaller scans should be performed or, if restoration-related not possible, it is recommended to take conventional impressions.

## Introduction

Today, conventional impressions (CI) and digital impressions (DI) are two ways to transfer the intraoral situation to the dental laboratory. A physical model is first produced on the basis of a CI. Dental restorations can then be fabricated directly on the gypsum model (conventional workflow) or the model is first scanned and then the dental restoration is digitally designed and milled (hybrid workflow).

In a completely digital workflow, the computer-aided-design and computer-aided-manufacturing process can be started directly after the DI has been taken with an intraoral scanner (IOS). In this way, dental restorations can be made in a single visit. In addition to saving materials, time, and costs, DI can also generate a high degree of standardization. DI can also avoid dimensional changes caused by deficiencies and properties of the impression material, errors in the processing or storage of impression materials, errors in the impression, and errors further down the process chain (disinfection, storage, transport, model fabrication). Depending on the system, IOSs add value by determining tooth shades, diagnosing caries, and analyzing preparations. However, there are high acquisition costs, limited connectivity among the different systems, and incompatibilities with scan bodies. Patients are more satisfied with DI than with CI, especially patients with strong gag reflexes, breathing difficulties, and taste sensitivities [1].

Dentists can expect a steep learning curve in terms of DI accuracy and scanning time. Training positively influences the accuracy and scanning time of complete-arch scans (CASs) [2, 3]. Dentists and dentistry students have different preferences when it comes to the impression method. Although dentists consider DI to be more effective, they still prefer CI whereas students prefer DI [4].

An impression method can only be deemed successful if the resulting dental restoration has a clinically acceptable fit. For single crowns and short-span fixed partial dentures (FPD), DI gives a comparable or better internal fit than CI does [5, 6]. Likewise, digital registration of the jaw relation appears to require no or at least fewer occlusal adjustments [7–10]. However, which impression method is better for creating a tooth-supported, jaw-spanning FPD is unknown. Advancements in the hardware and software of scanning systems has reduced the gap between CIs and DIs with regard to complete arch impressions. Recent studies have shown promising results for CASs made by IOSs [11, 12]. However, most of these studies were performed on fully dentate models [11, 13–17] or patients with defect-free or restoration-free teeth, [12, 18–23] which are not applicable to situations requiring prosthetic treatment. Therefore, it is necessary to compare the performance of DIs and CIs in partially edentulous dentition [24–26]. From a prosthetic point of view, it also makes sense to examine partially edentulous situations in which the abutment teeth are prepared. No such studies have been conducted to date.

The aim of this study was to compare the accuracy of CI with that of DI taken with two different IOSs in reproducing a partially edentulous dentition with complete-crown preparations. It was hypothesized that there would be no difference in accuracy between the three impression methods.

## Materials and methods

First, an upper jaw was virtually designed (computer-aided design (CAD) file) simulating the situation of a partially edentulous maxilla with central incisors, canines, and first and second molars as residual teeth. Except for the second molars, the teeth were given full-crown preparations, simulating a patient treated with a cross-arch fixed partial denture (FPD).

**Fig. 1:**
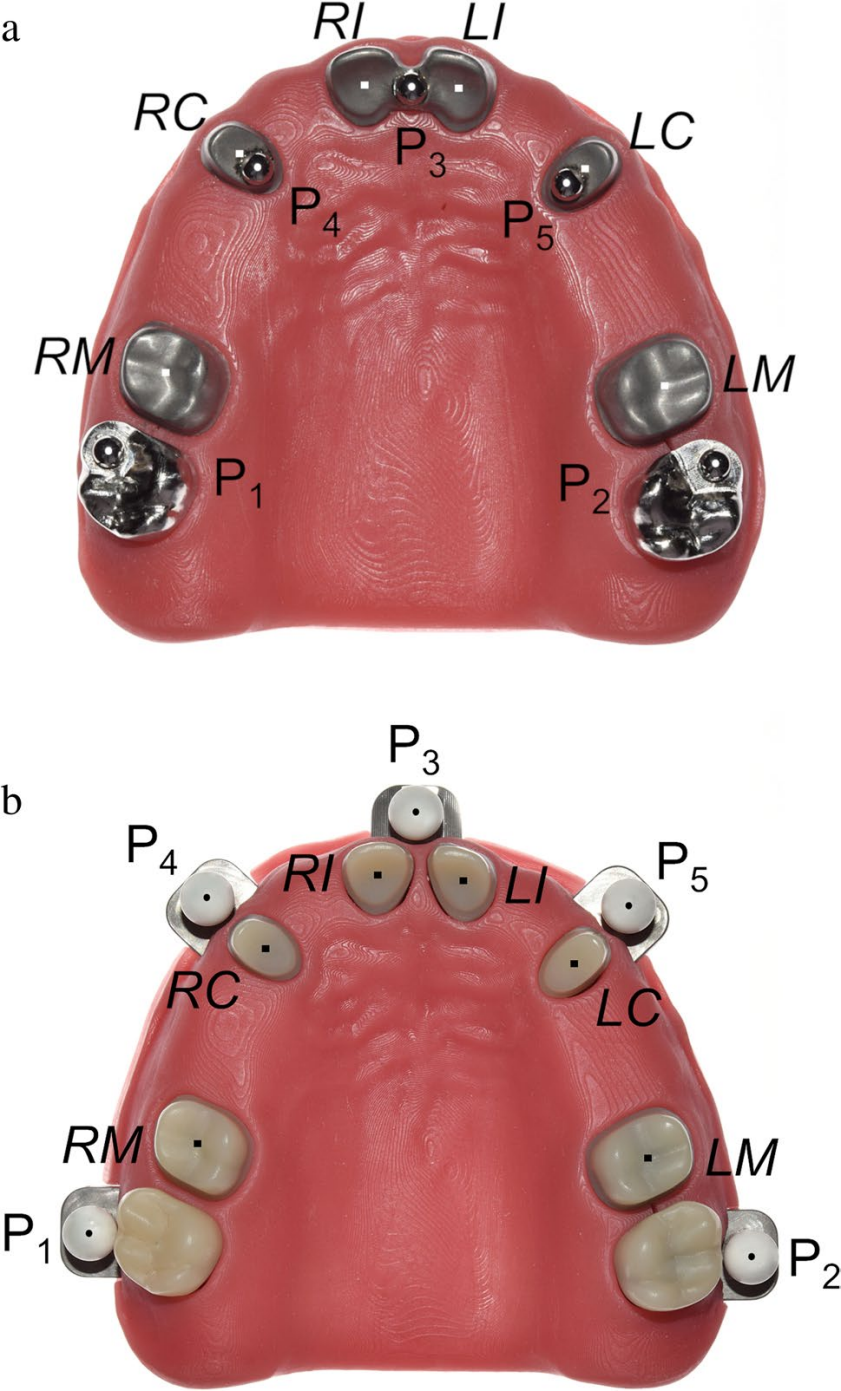
Reference models. Center points of prepared teeth (RM = right molar. RC = right canine. RI = right incisor. LI = left incisor. LC = left canine. LM = left molar) are marked with a square. Center points of each precision ball (P_1_–P_5_) are marked with a circle. **a** For conventional impression. **b** For digital impression

Second, precision balls respective for the impression method (CI or DI) were virtually added to this CAD file, resulting in two virtual reference models. For the virtual CI reference model (Fig. 1A), 5 precision balls (diameter = 3.175 mm) were placed occlusally to the second molars, the canines, and between the two central incisors. For the DI reference model (Fig. 1B), 5 precision balls (diameter = 5.911 mm) were placed buccally along the dental arch at the level of the margin lines of the second molars, canines, and the proximal contact of the central incisors. In each model, the five precision ball centers lay in a (horizontal) plane. These horizontal planes of the CI and DI model were in parallel.

Third, the metallic model base, shaped like a maxilla (Colado® CAD CoCr4, Ivoclar Vivadent AG), and the teeth were manufactured as separate parts. For the CI model (Fig. 1A), monolithic metal teeth made of cobalt-chromium alloy (Colado® CAD CoCr4. Ivoclar Vivadent AG) were used. The DI model (Fig. 1B) was designed in accordance with the specifications of E ISO 20896:2018–05. Therefore, surfaces were made of materials with optical properties close to those of natural teeth, i.e., the teeth consisted of a polymethyl methacrylate crown (SR Vivodent® CAD, Ivoclar Vivadent AG) placed on an alloy core (Colado® CAD CoCr4, Ivoclar Vivadent AG). Then, surfaces of all prepared teeth were digitized with 25 µm measurement grid distance and high precision (µscan select + with CLS4 sensor, NanoFocus AG; accuracy 0.32 µm) to create a digital reference data set of each tooth.

Forth, precision balls and teeth were welded to the model base in the planned position respective for the impression method, resulting in two physical (and almost identical) reference models. For the physical CI model, 5 stainless steel precision balls (diameter = 3.175 mm; G3; shape deviation tDWS/VDWS ≤ 0.08 µm, mean roughness value Ra ≤ 0.01 µm; variation of ball diameter VDWL ≤ 0.13 µm) were used. For the physical DI reference model, 5 ceramic precision balls (diameter: 5.9711–5.9714 mm; roundness deviation equatorial < 0.5 µm; TOPIC White, Saphirwerk AG), intended for use as calibration objects with light-optical measurement devices, were used. The metal pins on which the ceramic balls were mounted allowed a welded connection to the metal base. For both physical models, the metallic model base was covered with a stereolithographically produced gingival mask (FREEPRINT® gingiva, Detax GmbH & Co. KG). After the welding process measurements were made with a coordinate measuring machine (CMM, Mar-Vision 222; Mahr GmbH, accuracy < 1 − 2 µm) for both models to determine the spatial positioning of the welded precision balls and prepared teeth.

**Fig. 2:**
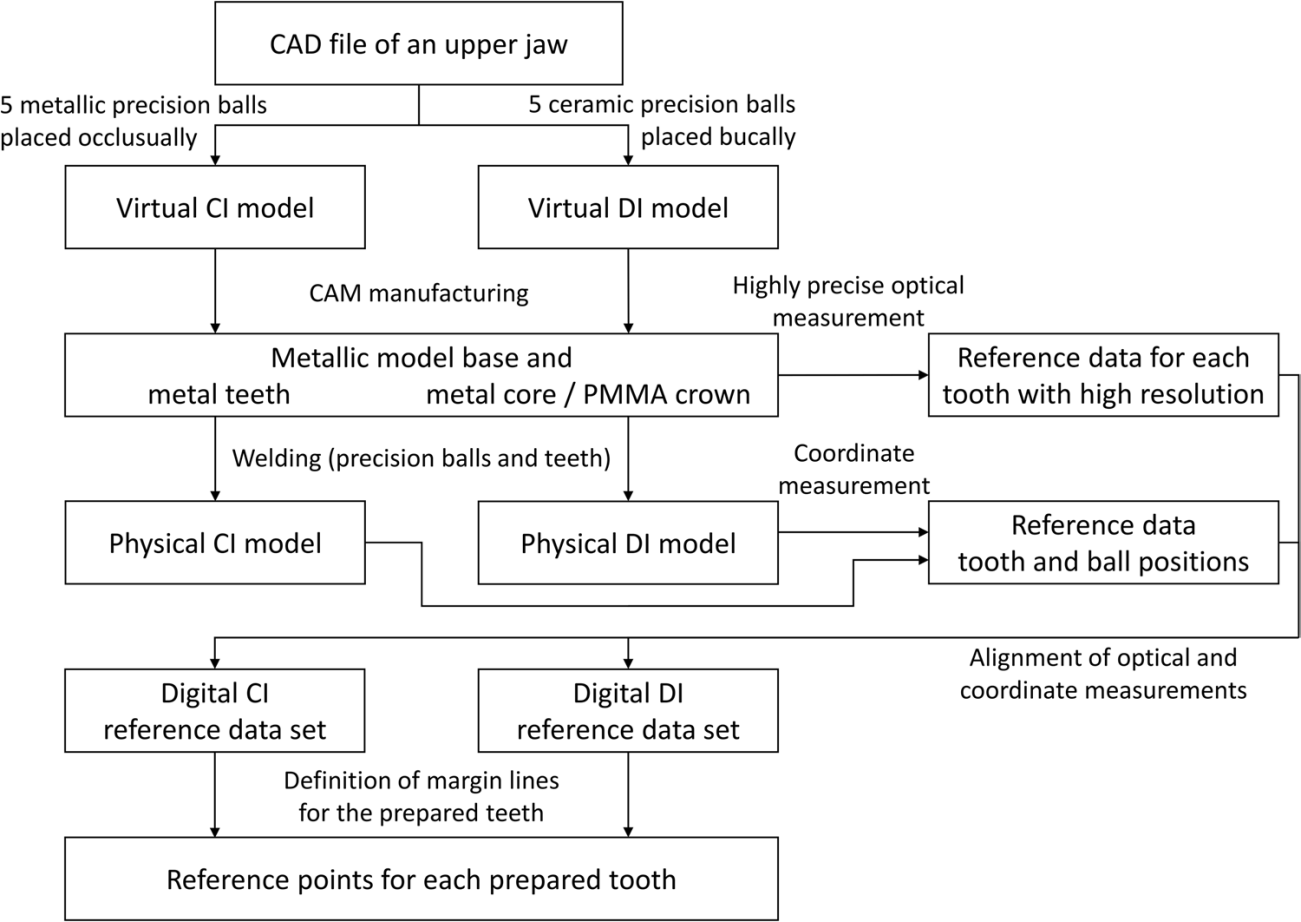
Schematic diagram of generating the reference data set

Fifth, in order to obtain a digital reference data set, the previously digitized teeth were globally aligned with the coordinate measurement of the complete model (Geomagic Design X, 3D Systems) each for both models. Precision ball centers were determined by minimizing the sum of all squared errors for any sphere with the given nominal radius and variable center position. For each reference data set, the five center points were numbered as shown in Figs. 1A (CI) and Fig. 1B (DI), with *P*_1_ in the region of the right second molar, *P*_2_ in the region of the left second molar, *P*_3_ in the region of the incisors, *P*_4_ in the region of the right canine, and *P*_5_ in the region of the left canine. A global coordinate system was defined as follows: *P*_1_ as origin, x-axis in direction *P*_1_*P*_2_, and xy-plane defined by *P*_1_, *P*_2_, and *P*_3_ with the y-axis oriented in the anterior direction. For the definition of the center points at margin level, the margin lines were first defined as splines (Geomagic Design X) on the high-resolution images of the prepared teeth. Then, the center points were gained by “extraction” which results in the centers of mass of the respective lines. A local coordinate system with axes parallel to those of the global coordinate system was added at the respective center point of the margin line of each prepared tooth, resulting in angles of 0 degrees between the corresponding axes in the reference cast. A schematic diagram of generating the reference data sets can be seen in Fig. 2.

The models were inserted in a phantom head, which was fixed onto a dental chair in a windowless room with artificial lighting to simulate clinical conditions during scans and CIs. The room temperature was 23.8 ± 0.4 °C and the relative humidity was 55.5 ± 2.3% for all scans. All DIs and CIs were performed by the same experienced investigator (MW).

For each IOS, 30 scans and for the CI, 30 impressions were taken from the respective reference model. The IOSs used were Omnicam AC (OC) (Dentsply Sirona) and Primescan (PS) (Dentsply Sirona). Scanning was performed according to the manufacturer’s instructions for a CAS (Table 1). Scans were postprocessed using the manufacturers’ software and were exported in standard tessellation language (STL) file format for further evaluation.

**Table 1:** Specifications for intraoral scanners

Intraoral scanner	Software	Scan sequence	Hardware settings
Omnicam	Sirona Connect v5.1.1	First scan quadrant: P (45° and 90°), O, B (45° and 90°) Second scan quadrant: P (90° and 45°), B (45° and 90°), O	CPU, Intel® Core™ i7-5820 K 3.30 GHz RAM, 32.0 GB Display card, AMD Radeon™ RX 470 Series
Primescan	Sirona Connect v5.1.1	P, O, B	CPU, Intel® Core™ i7-8700 3.20 GHz RAM, 32.0 GB Display card, AMD Radeon RX 570 Series

*O*, occlusal; *B*, buccal; *P*, palatal

The CI was performed using a dual phase impression technique with polyvinyl siloxane (PVS) material (Aquasil Ultra + XLV and Aquasil Ultra + Heavy, Dentsply Sirona). All CIs were removed from the model after 15 min, which is three times the clinical setting time for this PVS. The extended setting time at room temperature was necessary because shrinkage effects were observed 10 min after mixing in the pretests. The metallic Rimlock trays were individualized with a palatal stop and a dorsal dam to ensure a permanent distance between the tray and the teeth and positional stability during the setting time, and to support a seamless flow of the impression material to the tooth row. CIs were disinfected for 5 min (PrintoSept-ID, Alpro Medical GmbH) and then poured with type IV gypsum (esthetic-base gold, dentona AG) no earlier than 30 min after disinfection. The saw-cut models were digitized using a laboratory scanner (D2000, 3shape A/S) with quality control software to generate a digital data set in STL file format.

For all scans, including the gypsum model scans for the CI, the centers of the five precision balls (*P*_1′_–*P*_5′_, numbering as introduced for the reference model) were determined by optimization (method of least squares; squared deviations at the triangle corner points were weighted with proportionate surface area using MATLAB version R2020a, MathWorks). For the prepared teeth, each reference tooth surface together with its local coordinate system was aligned separately to the scan data using a best-fit algorithm (Geomagic Design X; 3D Systems). For each two teeth, distances between the transformed coordinate origins (located at the center of the margin line) and angles between the transformed vertical axes were determined and compared to the values recorded for the reference model. With 6 abutment teeth, there are 15 possible pairings. For better clinical interpretation, these pairings were categorized (Table 2). The 4 pairings RM-LI, RC-LI, RI-LC, and RI-LM were excluded from the evaluation since they do not meet to a common clinical treatment.

For each scan, a coordinate system was defined by *P*_1′_, *P*_2′_, and *P*_3′_ in an identical manner as for the reference data set and used to align scan and reference data set. Vertical distances (in z-direction) between each two tooth center points were measured as well and vertical distance deviations from the reference distance were calculated. In addition, point coordinates in the horizontal plane (xy-plane) were compared to the respective reference position and analyzed for further insight. Here, the coordinate origin was shifted to the starting point of the scan path, i.e., the center point of the right molar.

Thirty impressions of the reference model were taken for the CI, PS, and OC. To assess and compare the accuracy of the CI, PS, and OC, distance and tooth axis deviations from the reference model were calculated for 15 distances (each defined by two center points at the margin level). These were analyzed overall, i.e., over all considered dental arch/scan-path lengths and at the clinical category level. As part of the overall analysis, a linear mixed-effects model was fitted with the measure deviation as the dependent variable and the impression method (CI, PS, OC) as the independent variable. A random intercept was added to the model at the dental arch/scan path level to account for the repeated scan of each path. Furthermore, a one-way ANOVA *F*-test was performed using the fitted mixed-effects model to determine any differences between the impression methods at a 5% significance level. If the *F*-test revealed a significant difference, post hoc pairwise comparisons tests were performed to identify between which impression methods the significance difference was. The reported *p*-values of the pairwise comparisons were Tukey adjusted to account for the type I error inflation that occurs when multiple tests are performed.

The second analysis investigated whether the accuracy of the impression methods was influenced by the clinical category (crown block, short span FPD, long span FPD, quadrant, 2 sextant, anterior segment, cross arch) defined according to clinically relevant treatment situations. For this purpose, a linear mixed-effects model was fitted with measure deviation as the dependent variable and the impression method, clinical category, interaction between both, and a random intercept at the dental arch/scan-path level as the independent variables. A two-way ANOVA *F*-test was performed using the fitted mixed-effects model. If the *F*-test revealed a significant effect for the interaction term, post hoc pairwise comparisons tests were performed for each clinical category to determine between which impression methods the significant difference existed. The reported *p*-values of the pairwise comparisons were Tukey adjusted.

For further, purely descriptive, comparative analysis, the deviations of the 30 impressions in each group were first summarized for each distance and tooth axis, each categorized appropriately by the dental arch length (for CI, Table 2) or the scan-path length (for OC and PS, Table 2) by calculating the 50% and 90% quantiles.

**Table 2:** Clinical category and corresponding distances defined by center points at the margin level with respective dental arch length (for conventional impressions) or scan-path length (for digital impressions)

Clinical category	Distance	Number	Dental arch length	Scan-path length
			[mm]	
Crown block	RI–LI	30	9.3	8.9
Short span FPD	RI–RC	30	14.1	14.8
	LI–LC	30	15.2	15.3
Long span FPD	RC–RM	30	23.0	22.2
	LC–LM	30	22.4	22.3
Quadrant	RI–RM	30	37.1	37.0
	LI–LM	30	37.6	37.6
2 sextant	RM–LC	30	61.6	61.2
	RC–LM	30	61.0	61.3
Anterior segment	RC–RC	30	38.6	39.0
Cross arch	RM–LM	30	84.0	83.5

## Results

### Distance deviations

**Fig. 3:**
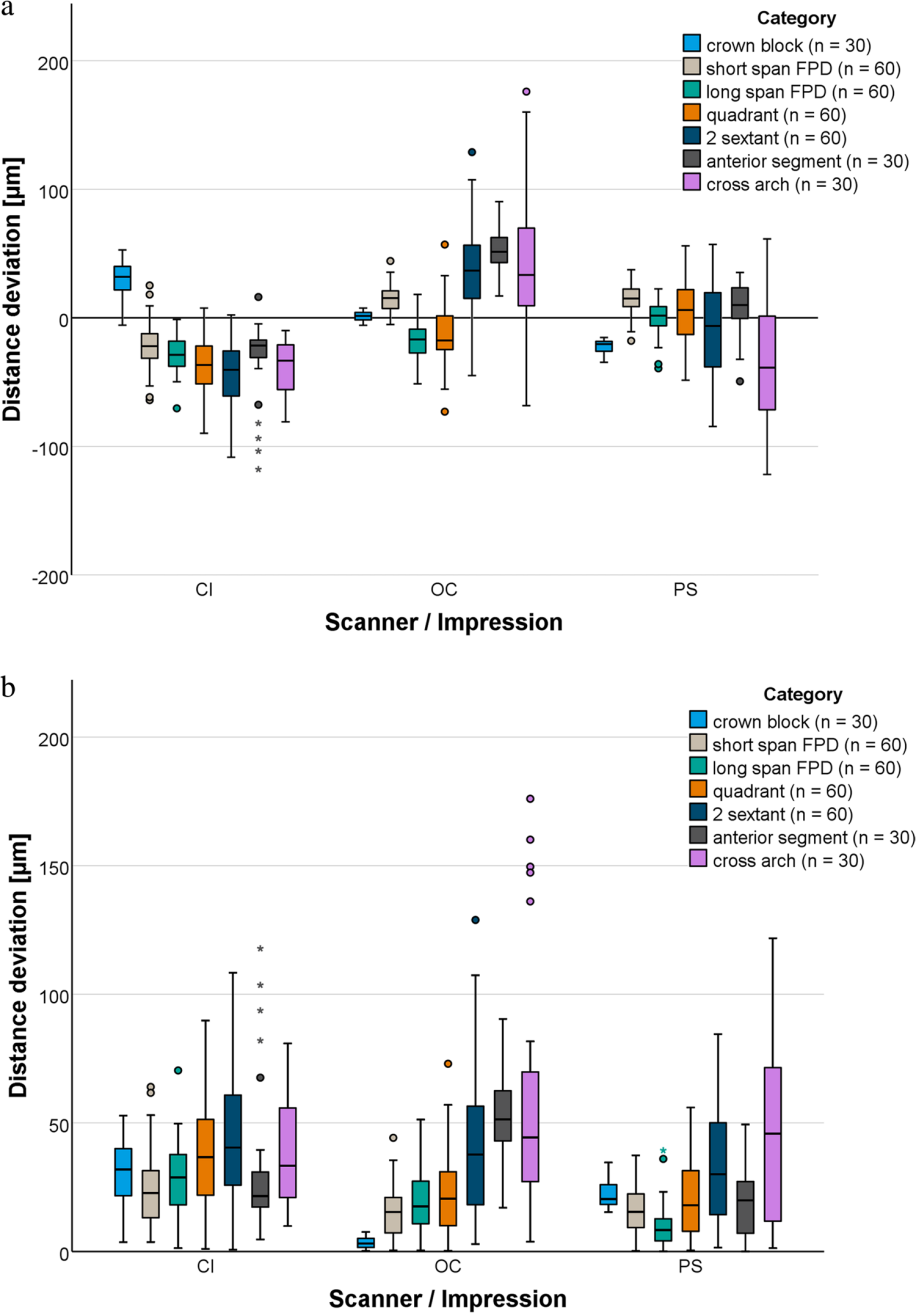
Total distance deviations pooled for clinical category for conventional impression (CI) and intraoral scanners (OC, Omnicam. PS, Primescan). **a** Signed distance deviations. **b** Absolute distance deviations

The signed total distance deviations are displayed in Fig. 3A. Both CI and the two IOS did not behave uniformly with respect to over- or underestimation of the reference distances. Device-specific deviation patterns were seen with DI. OC tended to overestimate the reference distances, while PS tended to underestimate them. Likewise, measured distances for CI were mainly shorter than the reference distances.

For DI, the largest deviations were 176 µm for OC and 122 µm for PS, and both occurred over the cross-arch distance (Fig. 3B and Table 3). For CI, the largest deviation was 118 µm and occurred over the anterior segment distance. Among the impression methods, larger mean absolute deviations were associated with one of the following four distances: the cross arch, the anterior segment, the 2 sextant distance, and the quadrant (Table 3). Mean absolute distance deviations of the crown block, the short-span FPD, and the long-span FPD were lower for both scanning systems compared to CI (Table 3).

**Table 3:** Absolute distance deviations [µm] between reference models and scans in relation to center points at the margin level per impression or intraoral scan (*CI*, conventional impression; *OC*, Omnicam; *PS*, Primescan) and clinical category

Category	CI					OC					PS				
	Mean	SD	Min	Max	Median	Mean	SD	Min	Max	Median	Mean	SD	Min	Max	Median
	[µm]														
Crown block	30	13	4	53	32	3	2	0	8	3	22	5	15	35	20
Short span FPD	24	14	4	64	23	15	9	0	44	15	16	9	0	37	15
Long span FPD	28	14	1	70	29	20	13	0	51	18	9	8	0	39	8
Quadrant	37	19	1	90	37	22	16	0	73	21	20	14	0	56	18
2 sextant	44	25	1	108	40	43	31	3	129	38	32	22	2	85	30
Anterior segment	33	29	5	118	22	53	16	17	90	51	19	12	0	49	20
Cross arch	39	21	10	81	33	59	48	4	176	44	47	34	1	122	46

*SD*, standard deviation; *Min*, minimum; *Max*, maximum

The vertical distance deviations are presented in Table 4. The maximum deviations were not concentrated in one clinical category, but were specific for the impression method. The largest vertical deviations occurred with PS, followed by OC and CI.

**Table 4:** Vertical distance deviations [µm] between reference models and scans in relation to center points at the margin level per impression or intraoral scan (*CI*, conventional impression; *OC*, Omnicam; *PS*, Primescan) and clinical category

Category	CI					OC					PS				
	Mean	SD	Min	Max	Median	Mean	SD	Min	Max	Median	Mean	SD	Min	Max	Median
	[µm]														
Crown block	5	4	0	20	4	17	10	1	52	15	15	8	2	29	16
Short span FPD	11	9	0	47	11	16	10	1	37	14	24	18	0	71	23
Long span FPD	11	9	0	43	8	28	23	0	86	23	13	9	1	39	13
Quadrant	8	5	0	22	7	26	13	2	67	27	16	11	0	36	14
2 sextant	10	9	0	43	7	27	22	1	76	21	49	20	5	106	50
Anterior segment	14	10	0	40	13	38	25	1	81	33	54	25	4	112	54
Cross arch	2	1	0	4	1	4	4	1	13	2	43	11	27	68	42

*SD*, standard deviation; *Min*, minimum; *Max*, maximum

Regarding total distance deviations, PS performed best (*p* < 0.001), followed by OC (*p* = 0.06) and CI. Regarding vertical distance deviations, CI performed best (*p* < 0.001), followed by OC (*p* < 0.001) and PS.

Pairwise comparisons of the accuracy of the impression methods at each clinical category showed that PS was superior to CI in all categories except for cross arch. The long span FPD, quadrant, 2 sextant, and anterior segment differed significantly between CI and PS (*p* ≤ 0.01). In contrast, there were no statistically significant differences for crown block, short span FPD, and cross arch (*p* ≥ 0.06). OC was superior to CI in all categories except for the anterior segment and cross arch. There were significant differences in all clinical categories between CI and OC (*p* ≤ 0.03) except for long span FPD and 2 sextant (*p* ≥ 0.08). Finally, PS was superior to OC for all categories except for crown block and short span FPD. There were significant differences between PS and OC in crown block, long span FPD, 2 sextant, anterior segment, and cross arch (*p* ≤ 0.05).

For vertical distance deviations, nearly all pairwise comparisons between the different scan-path categories were statistically significant (*p* ≤ 0.0083). Exceptions were the crown block between PS and OC (*p* = 0.92), the short-span FPD between CI and OC (*p* = 0.12), the long-span FPD between CI and PS (*p* = 0.56), and the cross arch between CI and OC (*p* = 0.8).

**Fig. 4:**
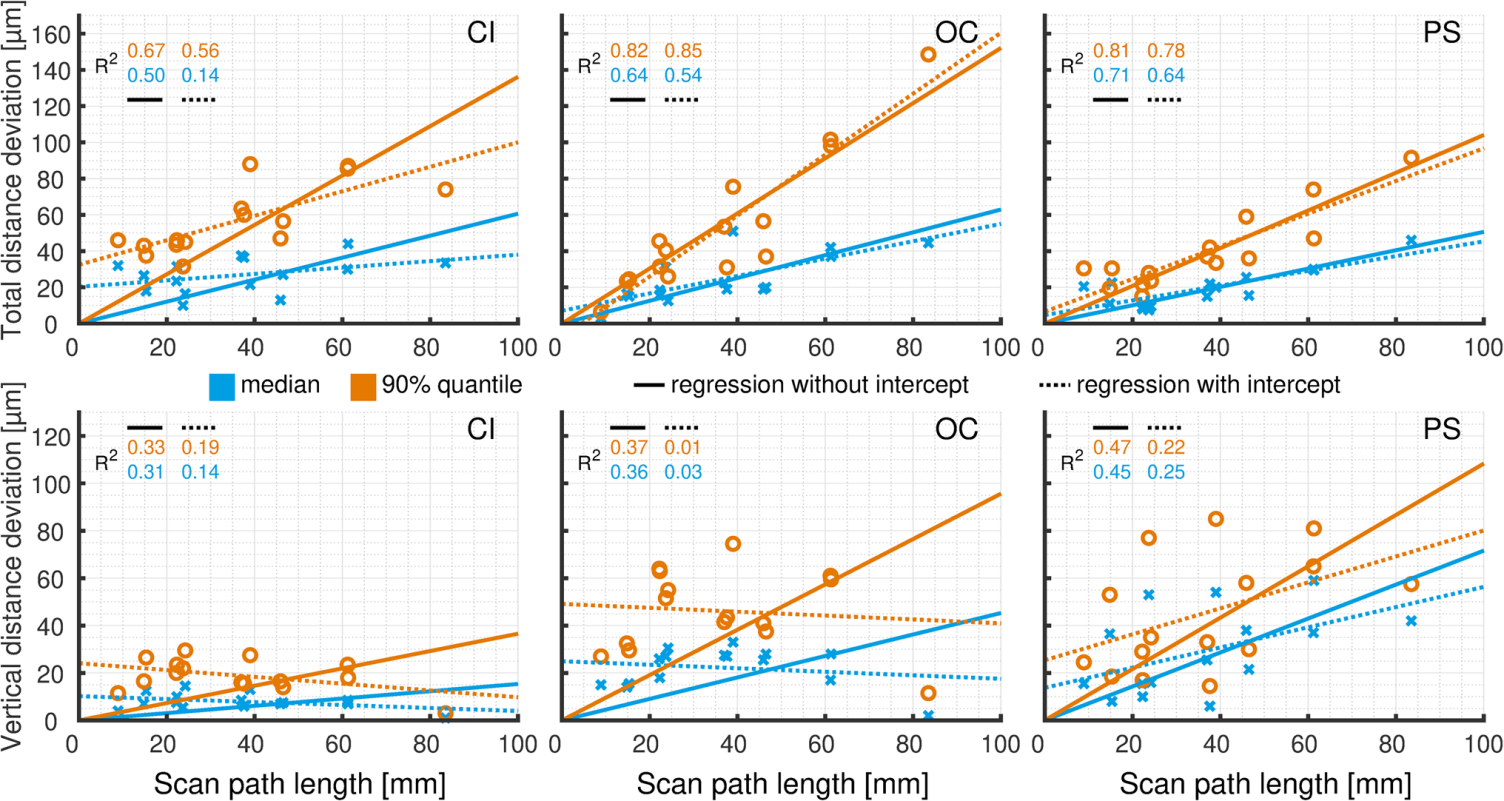
Total and vertical distance deviations for conventional impression (CI) and intraoral scanners (OC, Omnicam. PS, Primescan) displayed for 50% and 90% quantile values

Figure 4 shows the regression lines of the 50% and 90% quantiles for the total and vertical distance deviations. For PS and OC, regression lines with and without intercept showed almost perfect agreement for the total distance deviations. Further analysis of the regression lines revealed a good linear relationship between scan-path length and total distance deviations. For CI, no agreement was found between the regression lines with and without intercept. These data showed only a slight dependence of the scan path length and were not approximated well when neglecting the intercept. No consistent relationship was detected between dental arch length or scan-path length and vertical distance deviations. There was a high degree of dispersion for PS and OC while there was almost uniform variation across all clinical categories for CI.

### Tooth axis deviations

Impression inaccuracies led to deviations of the tooth axes (Table 5). Deviations were the smallest for the CI (*p* < 0.001) followed by PS (*p* < 0.001) and OC. All pairwise comparisons of tooth axis deviations were statistically significant (p ≤ 0.05), except for the 2 sextant (*p* = 0.06) and the anterior segment (*p* = 0.87) between CI and PS.

**Table 5:** Tooth axis deviations [°] between two prepared teeth per impression or intraoral scan (*CI*, conventional impression; *OC*, Omnicam; *PS*, Primescan) and clinical category

Category	CI					OC					PS				
	Mean	SD	Min	Max	Median	Mean	SD	Min	Max	Median	Mean	SD	Min	Max	Median
	[°]														
Crown block	0.24	0.11	0.05	0.42	0.23	0.10	0.04	0.01	0.17	0.11	0.31	0.03	0.25	0.36	0.30
Short span FPD	0.15	0.07	0.02	0.35	0.14	0.53	0.09	0.35	0.81	0.52	0.24	0.06	0.13	0.38	0.24
Long span FPD	0.15	0.07	0.01	0.35	0.15	0.37	0.20	0.06	0.84	0.31	0.26	0.08	0.06	0.39	0.26
Quadrant	0.18	0.08	0.04	0.36	0.19	0.54	0.14	0.17	0.81	0.54	0.40	0.10	0.23	0.62	0.39
2 sextant	0.21	0.08	0.05	0.45	0.20	0.95	0.21	0.44	1.25	0.98	0.26	0.12	0.03	0.51	0.26
Anterior segment	0.11	0.06	0.01	0.23	0.10	0.96	0.10	0.72	1.16	0.95	0.12	0.05	0.02	0.22	0.12
Cross arch	0.32	0.07	0.16	0.44	0.34	0.83	0.18	0.45	1.15	0.83	0.22	0.09	0.08	0.50	0.20

*SD*, standard deviation; *Min*, minimum; *Max*, maximum

**Fig. 5:**
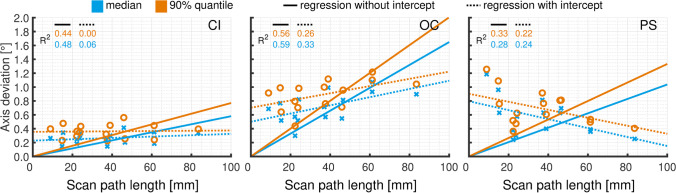
Tooth axis for conventional impression (CI) and intraoral scanners (OC, Omnicam. PS, Primescan) displayed for 50% and 90% quantiles

Figure 5 shows the regression lines of the 50% and 90% quantiles for tooth axis deviations. No relationship was detected between dental arch length or scan-path length and tooth axis deviations.

### Point deviations in the reference coordinate system

**Fig. 6:**
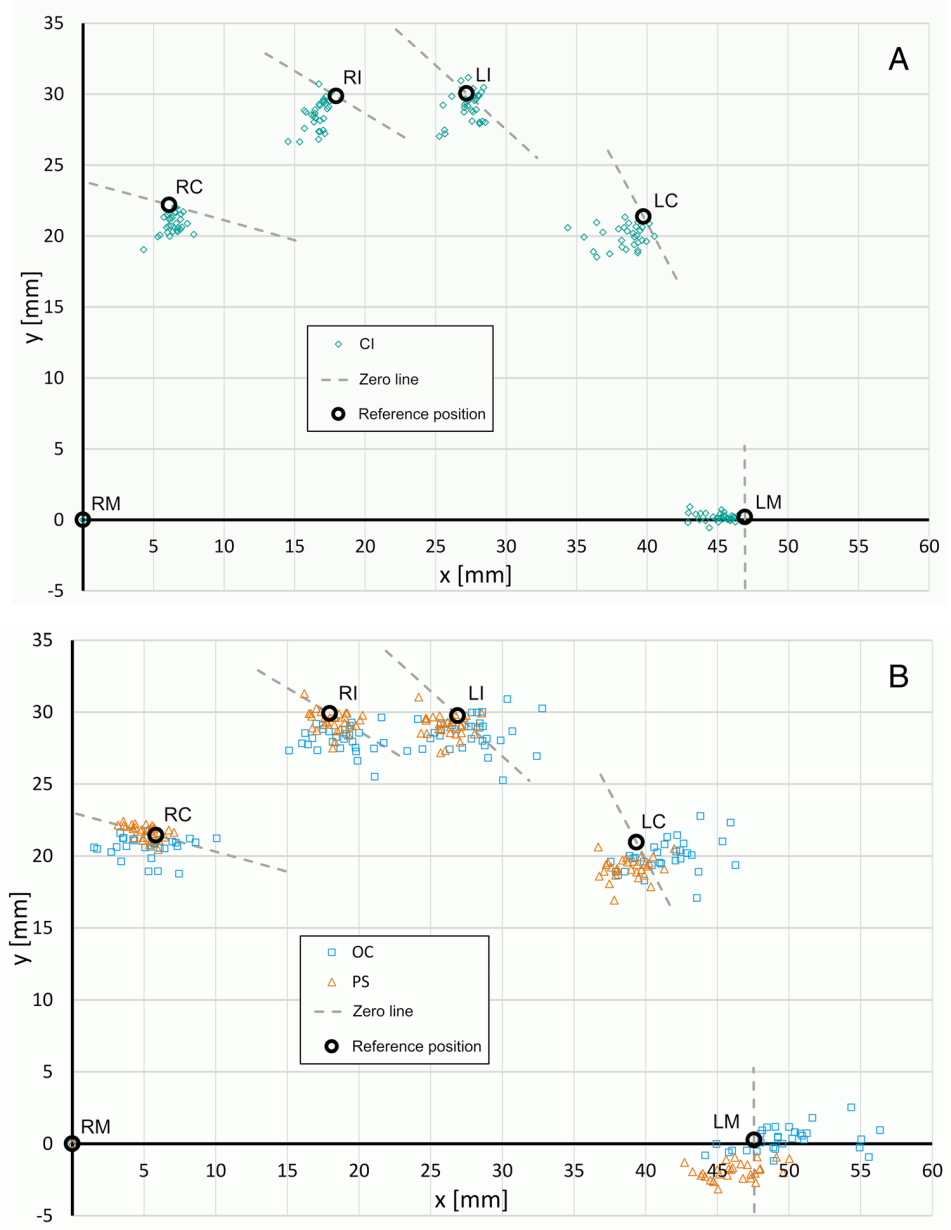
Horizontal deviations (with an enlargement factor of 50) of center points of prepared teeth (RM = right molar. RC = right canine. RI = right incisor. LI = left incisor. LC = left canine. LM = left molar) for conventional impression (CI) and intraoral scanners (OC = Omnicam. PS = Primescan). Circular lines (zero line (grey dashed line) with center RM and radius defined by reference distance to RC, RI, LI, LC, or LM state point deviations in the scans for which no distance deviations occur. **a** Conventional impression. **b** Digital impression

Figure 6 shows the tooth center point coordinates in the horizontal plane. Since deviations from the respective reference positions were rather small, these differences were plotted with an enlargement factor of 50. The scattering of the tooth center points at margin level depended on the used impression method. With conventional impression taking, the distances were in general underestimated. Thus, it seemed that all center points lay on a shrunk dental arch. In contrast, tooth center points of DI deviated in all directions from the reference positions. When comparing the two intraoral scanning systems OC had a greater scatter than PS.

## Discussion

The aim of this study was to compare how accurately a partially edentulous dentition with full crown preparations was reproduced by CI and DI (DI using two different IOS, OC and PS). The null hypothesis that no statistically significant difference exists between the three impression methods was rejected.

There are numerous studies on impressions and scanning accuracy in the literature. However, most of these studies were conducted on fully dentate models [11, 17, 19] or in patients [12, 19, 21, 23, 27] with defect-free teeth, which do not represent typical cases requiring treatment. A few studies have dealt with the partially edentulous jaw [24, 25, 28, 29]. Mostly, however, with defect-free teeth and not in comparison to the currently valid gold standard for complete-arch impressions, the CI [24, 25, 28, 29]. Only one study has investigated the performance of IOSs in the partially edentulous maxilla [30]. The authors used three different IOSs (OC, PS, and TRIOS 4) on the same model and evaluated scanning accuracy by measuring distances between two center points of precision balls distributed around the dental arch followed by the respective deviation to the reference distance. The OC and PS in that study used the same software with the identical software version. And yet the comparison with the results of the present study shows that the value ranges of the scan inaccuracies shifted (no longer a main overestimation, but arranged around the zero point) and their scatter decreased.

A recent rapid umbrella review comparing the accuracy of digital and conventional impressions showed that DI is comparable to CI for single-unit up to three-unit fixed dental reconstructions [31]. For clinical scenarios involving long-span areas, CI was superior to DI [31]. In line with this review, the results on total distance deviations showed that DI can reproduce short and medium distances up to one quadrant with the same or even higher accuracy than CI does. The DI appears to have similar mean or median values of larger segments to the CI. However, with IOSs, scatter increases markedly for scan distances of one quadrant or diagonally or across the palate compared with CI. The 3D data set of the intraoral situation generated by the IOS is created by lining up individual images through an alignment and stitching process. Each step is subject to error, which accumulates with increasing scan distances [32]. Resulting in the largest deviations measured over the cross-arch distance [14, 15, 17, 30, 33]. Although hardware and software developments have greatly improved scanning accuracy [11, 34], scatter is still much higher with DI than with CI. This seems to be particularly critical with regard to the possible fit of FPDs, as dentists want repeatable results in order to avoid expensive and time-consuming new fabrications for both patient and dentist. For a better assessment of the possible range of indications, it is therefore useful to include the outliers, in particular the 90% quantile values, in addition to the comparison of the mean values.

For the CI, the anterior segment was associated with the greatest impression inaccuracies. Compared to all other clinical categories examined, the largest scatter was observed here. The cause could be deformations occurring during demolding in the anterior region, which may exceed the recovery possibilities of the PVS.

Vertical and tooth axis deviations were also evaluated in this study. In cases with more than two abutment teeth, vertical differences between the intraoral position and the position in the scan result in larger marginal gaps on at least one tooth. Crowns require a marginal gap of ≤ 120 µm [35]. Since deviations perpendicular to the measurement direction have almost no effect on the measured values, vertical distance deviations cannot be represented by means of approximately horizontally measured distances alone and need to be considered separately. For example, a deviation of 100 µm in the z-direction affects the total length of all distances by an average deviation of only 1.4 ± 0.9 µm (min: 0.1 µm, max: 3.3 µm). The CI was superior to the IOS in terms of vertical and tooth axis deviations; vertical deviations of more than 100 µm were found in the DI at large distances.

An interpretation of the impression and scanning inaccuracies found is difficult due to the lack of a threshold value for total and vertical deviations or for tooth axis deviations. Jaw-spanning restorations based on CI are clinically successful, so conclusions about the applicability of DI can only be based on comparisons with CI. Prosthesis fit is not only dependent on the impression but also adjustable within certain limits during the manufacturing process. However, only an accurate reproduction of the intraoral situation can lead to the best possible result.

A limitation of this study is the use of two reference models, each specific for the respective impression method. The model base and the anatomy of the teeth are identical for both models. Before the CMM, the teeth were welded onto the metal base. The welding process resulted in minor deviations of the teeth from the planned positions. Also, the individual definition of the margin lines and corresponding center points will cause minor differences between the two reference data sets. However, when comparing respective distances in the two reference data sets, deviations were 0.36 ± 0.22 mm. Changes in distance (or scan-path length) will affect the absolute distance deviation values observed in the experiments. Maximum slopes seen in Fig. 3 (total distance deviation: 1.6 µm/mm, vertical distance deviation: 1.2 µm/mm) and Fig. 4 (angular deviation 0.02°/mm) provide information about error propagation. For all distances, differences between the models were below 0.8 mm. Therefore, maximum errors for the 90% percentile values correlating with CI and DI model differences can be estimated to be < 1.3 µm for total distance deviation, < 1.0 µm for vertical distance deviation, and < 0.016° for axis deviations. These model-based errors are small compared to the observed inaccuracies. Furthermore, z-axes defined by the horizontal planes given by each three precision ball centers will differ to a small amount between both, the scans and the physical reference models. These deviations were, however, smaller than Δ*α* = 2°. Vertical length measurements are affected by a change in z-direction with the factor cos(Δ*α*). This leads to relative measurement errors less than 1-cosΔ*α* = 0.06%. With coordinate differences in z-direction of less than 1 mm, absolute errors were below 1 µm. With this knowledge, the authors believe that a direct comparison of data based on the two models is possible and permissible.

## Conclusions

Based on these findings of this study following conclusions were drawn:

1) The impression method affected the accuracy of impressions of partially edentulous maxilla with prepared teeth.

2) DI can be recommended for scans up to a quadrant. For larger scan volumes, the scatter of accuracy values increases and is therefore unsuitable for the fabrication of FDPs.

3) Vertical deviations are lower in CI than in DI.
